# Pneumonic Plague in a Dog and Widespread Potential Human Exposure in a Veterinary Hospital, United States

**DOI:** 10.3201/eid2504.181195

**Published:** 2019-04

**Authors:** Paula A. Schaffer, Stephanie A. Brault, Connor Hershkowitz, Lauren Harris, Kristy Dowers, Jennifer House, Tawfik A. Aboellail, Paul S. Morley, Joshua B. Daniels

**Affiliations:** Colorado State University, Fort Collins, Colorado, USA (P.A. Schaffer, S.A. Brault, C. Hershkowitz, L. Harris, K. Dowers, T.A. Aboellail, P.S. Morley, J.B. Daniels);; Colorado Department of Public Health and Environment, Denver, Colorado, USA (J. House)

**Keywords:** plague, pneumonic plague, dog, canine, Yersinia pestis, bacteria, human exposure, veterinary teaching hospital, vector-borne infections, zoonoses, Colorado, United States

## Abstract

In December 2017, a dog that had pneumonic plague was brought to a veterinary teaching hospital in northern Colorado, USA. Several factors, including signalment, season, imaging, and laboratory findings, contributed to delayed diagnosis and resulted in potential exposure of >116 persons and 46 concurrently hospitalized animals to *Yersinia pestis.*

Plague is rare in dogs, even in areas to which *Yersinia pestis* is endemic (*1**,**2*). We describe a case of canine pneumonic plague that resulted in >116 potential human exposures.

## The Study

**Figure 1:**
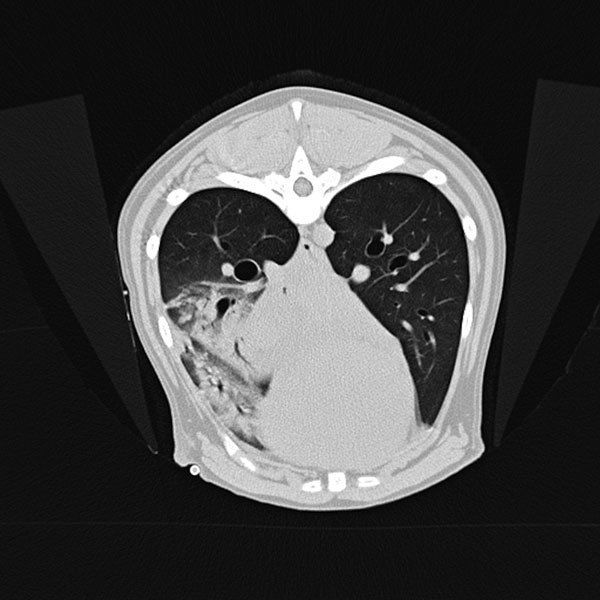
Transverse computed tomography of dog with pneumonic plague on day 2 of hospitalization, Colorado, USA. Image shows accessory lung lobar consolidation.

A 3-year-old mixed-breed dog was brought to a veterinarian in Colorado, USA, during December 2017 for evaluation of lethargy and fever 4 days after the dog was observed sniffing a dead prairie dog. Treatment with amoxicillin/clavulanic acid was initiated before referral the next day to the Colorado State University Veterinary Teaching Hospital (CSU-VTH; Fort Collins, CO, USA) because of progressive illness and development of hemoptysis. Imaging demonstrated unilateral lung lobar consolidation and small foci of parenchymal density in other lobes and intrathoracic lymphadenopathy (Figure 1).

**Figure 2:**
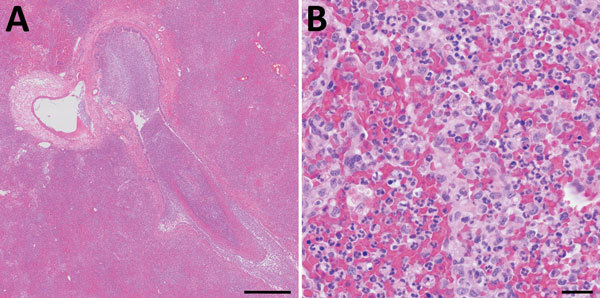
Histopathologic analysis of accessory lung lobe of dog with pneumonic plague (hematoxylin and eosin stain), Colorado, USA. A) Parenchyma, which is diffusely effaced by necrohemorrhagic pneumonia. Scale bar indicates 500 μm. B) Alveolar detail, which is obscured by necrosis, hemorrhage, and suppurative inflammation without intralesional bacteria. Scale bar indicates 20 μm.

Plague was considered unlikely because of the animal species, season, lack of peripheral lymphadenopathy, and unilateral lobar imaging pattern consistent with an aspirated foreign body, which is common in dogs (*3*). Treatment with ampicillin/sulbactam and enrofloxacin was initiated, and accessory lung lobectomy was performed to remove the presumed source of sepsis. Consolidation of the accessory lobe and scattered dark red foci in other lung lobes were noted intraoperatively. Histologically, the excised lobe was effaced by severe necrosuppurative pneumonia with hemorrhage and fibrinous pleuritis but no intralesional bacteria (Figure 2).

After 48 hours of aerobic incubation, a swab specimen of lung parenchyma yielded light and pure growth of bacteria that we identified by using matrix-assisted laser desorption/ionization time-of-flight mass spectrometry (Vitek-MS, https://www.biomerieux.com) with 91.1% confidence as *Yersinia pseudotuberculosis*, although the database of the instrument contained mass spectra for 5 *Y. pestis* strains. Because signs were not consistent with *Y. pseudotuberculosis*, there was concern about misidentification. We performed PCR of the isolate the next day (5 days after admission) by using a Centers for Disease Control and Prevention (Atlanta, GA, USA) Laboratory Response Network protocol for *Y. pestis* (https://www.epa.gov/homeland-security-research/sam-and-us-centers-disease-control-and-prevention-cdc-laboratory-response).

The dog was humanely killed the same day because of progression of pneumonia and poor prognosis. A limited necropsy was performed by informed personnel and found diffuse necrosuppurative and hemorrhagic pneumonia and severe necrotizing tonsillitis. Only liver tissue was positive for *Y. pestis* by PCR.

The dog had been transported throughout the hospital and housed in an oxygen cage vented to the room, potentially exposing personnel from multiple clinical services. Those handling specimens in the diagnostic laboratory were also considered exposed to *Y. pestis*. Exposures during the first 2 days of hospitalization were considered most critical because fluoroquinolone treatment was initiated after admission, and guidelines from the Colorado Department of Public Health and Environment (CDPHE) state that veterinary patients with *Y. pestis* have limited contagious risk after 48 hours of appropriate therapy (*4*).

While PCR results were pending, paper sheets were circulated to personnel to record contact with the dog. After the positive PCR result, emails were sent to these persons, followed by emails to all personnel. The delay between suspicion and diagnosis of *Y. pestis* resulted in word of mouth traveling faster than official communication, which caused anxiety among personnel. Many expressed frustration that suspicion and diagnosis of plague did not occur earlier. Two hospital-wide meetings were held for questions, discussion, and feedback. An online postincident survey was conducted, and 52 respondents indicated that they were aware of their potential exposure within 48 hours of the diagnosis.

We found 116 documented potential human exposures (Table 1). CDPHE recommendations were based on risk assessments, and interventions were decided by potentially exposed persons in consultation with healthcare providers (Table 2). In addition, 46 hospitalized animals co-housed in the same room were classified as potentially exposed. Prophylactic antimicrobial drugs were recommended because most of these animals were critically ill and had decreased immune status. To our knowledge, there were no cases of *Y. pestis* infection in potentially exposed humans or animals. A fever developed in 1 person, but this fever was not determined to be caused by *Y. pestis* infection. One survey respondent reported adverse effects from antimicrobial drugs.

**Table 1:** Number of potentially exposed persons by occupation to dog with pneumonic plague, Colorado, USA*

Occupation	No. (%) persons
CSU-VTH employees	64 (55)
Veterinary students	35 (30)
Laboratory personnel	17 (15)
Total	116 (100)

*CSU-VTH, Colorado State University Veterinary Teaching Hospital.

**Table 2:** Public health risk–based recommendations and interventions for persons exposed to dog with pneumonic plague, Colorado, USA*

Level of interaction with dog	Recommended intervention	No. (%) contacts reporting intervention
Exposure (<6 feet) to dog with pneumonic plague; exposure to exudates, blood, or tissue without barrier precautions; bite or scratch from infected dog or flea	Antimicrobial drug prophylaxis	68 (59)†
Presence in critical care ward where dog was housed; exposure or contact after 48 h of appropriate patient antimicrobial drug treatment	Fever and symptom watch	38 (33)
Persons who did not meet the above criteria	No action (awareness education only)	Remainder of employees, staff, and students

*Ten (8%) of 116 listed contacts did not report chosen intervention. †Of respondents who specified type of antimicrobial drug therapy received, 37 received doxycycline only, 2 doxycycline and gentamicin, 1 doxycycline and ciprofloxacin, 1 doxycycline and trimethoprim/sulfamethoxazole, and 1 ciprofloxacin only.

## Conclusions

Several factors delayed the diagnosis of pneumonic plague, resulting in many potential exposures. Pneumonic plague is uncommon in dogs; most dogs with plague have bubonic or septicemic plague and signs of fever, lethargy, and peripheral lymphadenopathy (*1*). The occurrence of this case during December was outside the predominant period of plague transmission in the Northern Hemisphere (April–October) (*5*). Of 89 animals with plague reported to CDPHE during 2008–2017, only 1 case (in a wild lynx) occurred in December. The mild winter in Colorado during 2017 might have prolonged activity of flea vectors, consistent with climate models that predict altered plague seasonality (*6*). In humans, radiographic abnormalities typically include bilateral lobar changes (*7*); in this dog, the accessory lung lobe was primarily affected on initial imaging, and this finding was interpreted as aspiration pneumonia. Histologically, pneumonic plague usually results in acute necrosuppurative, hemorrhagic pneumonia with obvious colonies of bacteria (*8*). In this case, antibiosis might have resulted in the histologic absence of bacteria.

Matrix-assisted laser desorption/ionization time-of-flight mass spectrometry identified the isolate as *Y. pseudotuberculosis* with a high confidence score, but the species-level identification was considered suspicious. *Y. pestis* has been previously misidentified as *Y. pseudotuberculosis*, *Pseudomonas luteola*, and *Acinetobacter lwoffii* by automated systems (*9*) even when information for *Y. pestis* is present in databases. This isolate produced a strong peak at 3,061 m/z, which was present in spectra of the 5 *Y. pestis* isolates in the database and absent from *Y. pseudotuberculosis* spectra, likely corresponding to a *Y. pestis* biomarker previously described at 3,065 m/z (*10*).

At the time of this incident, CSU-VTH infection control standard operating procedures stressed suspicion of plague in cats and minimized the presence of this disease in dogs. Criteria for designating patients as high-risk plague suspects were opportunity for exposure (including geography and season), fever, and enlarged peripheral lymph nodes. Although the dog was febrile and had interacted with a prairie dog, the species, lack of peripheral lymphadenopathy, and nonseasonal presentation resulted in low-risk designation. Standard operating procedures are being updated to emphasize that dogs might be affected with plague year round and that lymphadenopathy might not be present. Isolation and plague testing for all dogs with pneumonia is not feasible, but increased suspicion of patients with hemoptysis might be appropriate. A relatively rare finding in dogs (*11*), hemoptysis was present in this dog, as well as in 2 other previously reported cases of canine pneumonic plague (*12**,**13*).

This unique case highlights the public health response challenges in a large teaching institution. Veterinary workers are at increased risk for infection with zoonotic diseases (*14*), and exposure to infectious agents is an occupational stressor with potential emotional toll (*15*). Veterinary hospital administrations should educate staff about zoonotic hazards, mitigate exposures, and communicate rapidly to personnel when potential exposures occur. The communication process in place at the CSU-VTH for zoonotic exposures has historically been used in small-scale events. The extensive potential exposures in this instance highlighted the shortcomings of the process for reaching large numbers of persons. These problems are being addressed through development of frequently updated email listservs and telephone lists. Computerized logs may also be useful for documenting contact with patients with historical and syndromic factors consistent with potential zoonoses.

In summary, pneumonic plague, although rare, should be considered in dogs that have fever and respiratory signs with potential exposure in disease-endemic areas, regardless of season and lobar distribution. Efficient zoonotic disease communication and response plans should be prepared for large-scale events.
